# Serum PCSK9 Levels Distinguish Individuals Who Do Not Respond to High-Dose Statin Therapy with the Expected Reduction in LDL-C

**DOI:** 10.1155/2014/140723

**Published:** 2014-07-17

**Authors:** Beth A. Taylor, Gregory Panza, Linda S. Pescatello, Stuart Chipkin, Daniel Gipe, Weiping Shao, C. Michael White, Paul D. Thompson

**Affiliations:** ^1^Department of Cardiology, Henry Low Heart Center, Hartford Hospital, 85 Seymour Street, Hartford, CT 06102, USA; ^2^Department of Health Sciences, University of Hartford, 200 Bloomfield Avenue, West Hartford, CT 06117, USA; ^3^Department of Cardiology, Hartford Hospital, 85 Seymour Street, Hartford, CT 06102, USA; ^4^Department of Kinesiology, University of Connecitcut, 2095 Hillside Road, Unit 1110, Storrs, CT 06269, USA; ^5^Department of Kinesiology, University of Massachusetts Amherst, 110 Totman Building, 30 Eastman Lane, Amherst, MA 01003, USA; ^6^Regeneron Pharmaceuticals, Inc., 777 Old Saw Mill River Road, Tarrytown, NY 10591, USA; ^7^University of Connecticut School of Medicine, 263 Farmington Avenue, Farmington, CT 06030, USA

## Abstract

The purpose of the present report was to examine whether proprotein convertase subtilisin/kexin type 9 (PCSK9) levels differ in individuals who do not exhibit expected reductions in low density lipoprotein cholesterol (LDL-C) with statin therapy. Eighteen nonresponder subjects treated with 80 mg atorvastatin treatment for 6 months without substantial reductions in LDL-C (ΔLDL-C: 2.6 ± 11.4%) were compared to age- and gender-matched atorvastatin responders (ΔLDL-C: 50.7 ± 8.5%) and placebo-treated subjects (ΔLDL-C: 9.9 ± 21.5%). Free PCSK9 was marginally higher in nonresponders at baseline (*P* = 0.07) and significantly higher in atorvastatin responders after 6 months of treatment (*P* = 0.04). The change in free PCSK9 over 6 months with statin treatment was higher (*P* < 0.01) in atorvastatin responders (134.2 ± 131.5 ng/mL post- versus prestudy) than in either the nonresponders (39.9 ± 87.8 ng/mL) or placebo subjects (27.8 ± 97.6 ng/mL). Drug compliance was not lower in the nonresponders as assessed by pill counts and poststudy plasma atorvastatin levels. Serum PCSK9 levels, both at baseline and in response to statin therapy, may differentiate individuals who do versus those who do not respond to statin treatment.

## 1. Introduction

Proprotein convertase subtilisin/kexin type 9 (PCSK9) modulates low density lipoprotein cholesterol (LDL-C) concentrations by binding to hepatic LDL receptors, facilitating their catabolism [1], thereby increasing circulating LDL-C. Statin therapy increases serum PCSK9 levels [2], a finding that may explain the nonlinear relationship between statin dose and LDL-C reduction and the variable response that patients show to statin therapy. The present analysis examined PCSK9 levels in subjects treated with 80 mg atorvastatin for 6 months who did not respond to statin therapy with the expected reduction in LDL-C to determine whether an exaggerated increase in circulating PCSK9 levels with statin therapy could explain blunted statin efficacy.

## 2. Materials and Methods

Eighteen subjects who completed the double-blind, randomized clinical trial, the Effect of Statins on Muscle Performance (STOMP; National Heart, Lung, and Blood Institute 5R01HL081893, NCT00609063 [3]), but did not exhibit the expected reduction in LDL-C with 80 mg atorvastatin treatment for 6 months (mean change ± standard deviation: 2.6 ± 11.4% reduction in LDL-C for atorvastatin nonresponders) were compared to 18 matched, atorvastatin-treated subjects who decreased LDL-C by 50.7 ± 8.5% over 6 months (atorvastatin responders), as well as 18 matched placebo-treated subjects (LDL-C increased 9.9 ± 21.5% over 6 months). Subjects were matched for age (29 ± 13 years), gender (8 males/group), BMI (25 ± 5 kg/m^2^), and baseline LDL-C (104 ± 29 mg/dL). Compliance to study drug was measured by pill counts of unused medication at 3 and 6 months as well as analysis of plasma atorvastatin at the posttreatment study visit. Medication compliance was higher in nonresponders than in the responder and placebo groups (98 ± 9% versus 94 ± 6% versus 94 ± 6%, resp.; *P* < 0.05). Furthermore, atorvastatin metabolites were nonsignificantly higher in atorvastatin nonresponders than responders (10 ± 20 ng/mL versus 8 ± 10 ng/mL; *P* = 0.70) and placebo (0 ± 0 ng/mL, *P* = 0.06).

Both total PCSK9 (which circulates in association with LDL particles by interacting with apoB100) and free PCSK9 in archived, frozen serum taken from fasting samples at baseline and after 6 months of treatment were measured using specific enzyme-linked immunosorbent assays (ELISA), proprietary to Regeneron Pharmaceuticals, Inc. (Tarrytown, NY), with reference being a recombinant full length human PCSK9. For the total PCSK9 assay, an acid treatment of the serum samples was included prior to analysis in order to dissociate PCSK9 complexes that might be present in the serum. PCSK9: alirocumab and PCSK9: LDLR complexes are present in the serum and both active and furin cleaved PCSK9 were measured by ELISA. One-way ANOVAs were used to compare baseline characteristics and change scores between groups, and a repeated measures analysis with group as the between-subjects factor and time as the within-subjects factor was used to compare changes in variables (PCSK9 and LDL-C) before and after study.

## 3. Results

**Figure 1:**
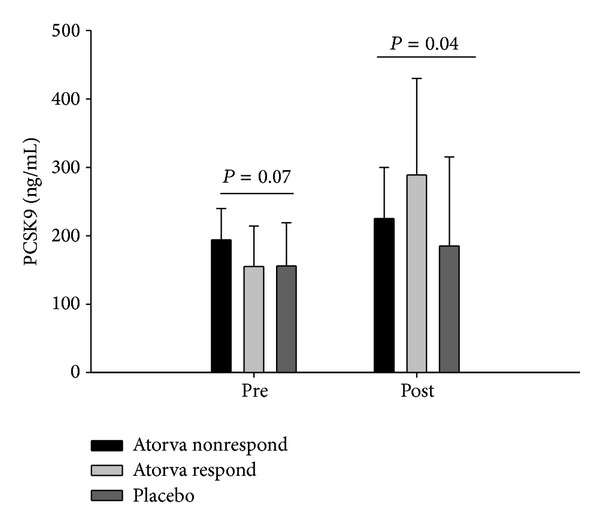
Free PCSK9 levels (group means ± standard deviations) before (pre) and after (post) 6 months of atorvastatin 80 mg or placebo treatment in 18 individuals who did not reduce LDL-C on atorvastatin (Atorva nonrespond) versus 18 individuals who did reduce LDL-C on atorvastatin (Atorva respond) and individuals on placebo (Placebo). *P* values indicate group differences at each time point.

Free PCSK9 (Figure 1) was marginally higher in atorvastatin nonresponders at baseline (*P* = 0.07) and significantly higher in atorvastatin responders after 6 months of treatment (*P* = 0.04). In addition, the change in free PCSK9 over 6 months with statin treatment was higher (*P* < 0.01) in atorvastatin responders (134.2 ± 131.5 ng/mL post- and prestudy) than in either the nonresponders (39.9 ± 87.8 ng/mL) or placebo subjects (27.8 ± 97.6 ng/mL). Total PCSK9 values at 6 months, as well as the change from baseline to 6 months, demonstrated a parallel trend (higher values and a greater change observed in atorvastatin responders), but these differences were not significant (*P* = 0.11 and 0.14, resp.), a finding that is likely attributable to the lower percentages of total (~30%) versus free (~70%) PCSK9 that circulate in the plasma. Finally, the change in LDL-C over 6 months was inversely correlated to the change in free PCSK9 (Pearson correlation = −0.31; *P* = 0.03) demonstrating that the greatest reductions in LDL-C were associated with the greatest increases in PCSK9.

## 4. Discussion

Our data indicate that patients whose reduction in LDL-C after 6 months of atorvastatin treatment was <20% of baseline level and who were classified as nonresponders had marginally higher free PCSK9 at baseline and did not increase free PCSK9 with atorvastatin treatment. This could simply be noncompliance with statin drug treatment, but this would not explain baseline differences in free PCSK9. Also, compliance was measured both by pill count, which was better over 6 months in atorvastatin nonresponders than in responders or placebo subjects, and by posttreatment plasma atorvastatin levels, which were nonsignificantly higher in nonresponders than responders. Without directly observing pill ingestion, we cannot exclude the possibility that nonresponders had only taken their medication shortly before the visit (thus not leaving sufficient time for atorvastatin to reduce cholesterol levels), but this seems unlikely to have occurred repeatedly throughout the nonresponder group.

Statins upregulate expression and secretion of PCSK9 by activating the sterol regulatory element-binding protein-2 (SREBP-2). Higher levels of PCSK9 decrease the number of hepatic LDL receptors and can produce hypercholesterolemia [4, 5]. Previous studies have noted that sensitivity to statin therapy is increased in mice and humans with minimally expressed levels of PCSK9 [6, 7] and that there is a correlation between the LDL-C lowering effects of statin therapy and change in PCSK9 levels [8, 9]. The hypothesis from collective data is that inhibition of cholesterol synthesis by statin therapy upregulates production of PCSK9. Consequently, PCSK9 inhibition is an emerging pharmacological target for patients with familial hypercholesterolemia and/or high levels of LDL-C who do not achieve sufficient lipid lowering with statin therapy. Here, for the first time, we demonstrate that baseline serum PCSK9 levels and the response to statin therapy may differ between those who do and those who do not respond to statin treatment. These findings support the converse of previous findings: lack of sensitivity to statin therapy in healthy adults may be predicted by higher levels of baseline PCSK9, although an abnormally high PCSK9 response to statin therapy does not appear to be further associated with the lack of response to statins. These preliminary results with a small sample need to be verified in additional studies, especially given that there are multiple gain of function and loss of function variants of PCSK9 which influence plasma LDL-C levels. However, the present data indicate that PCSK9 levels may be a useful clinical biomarker with which to predict or confirm diagnosis of the approximate 4–10% of individuals [10] who do not exhibit the expected reduction in LDL-C with statin therapy.
